# CARM1 Mediates Modulation of Sox2

**DOI:** 10.1371/journal.pone.0027026

**Published:** 2011-10-28

**Authors:** Hai-yong Zhao, Yan-jun Zhang, Hui Dai, Ye Zhang, Yu-fei Shen

**Affiliations:** National Laboratory of Medical Molecular Biology, Department of Biochemistry and Molecular Biology, Institute of Basic Medical Sciences, Chinese Academy of Medical Sciences & Peking Union Medical College, Beijing, China; University of Hong Kong, Hong Kong

## Abstract

Sox2 is a key component of the transcription factor network that maintains the pluripotent state of embryonic stem cells (ESCs). Sox2 is regulated by multiple post-translational modifications, including ubiquitination, sumoylation, acetylation and phosphorylation. Here we report that Sox2 is in association with and methylated by coactivator-associated arginine methyltransferase 1 (CARM1), a protein arginine methyltransferase that plays a pivotal role in ESCs. We found that CARM1 facilitates Sox2-mediated transactivation and directly methylates Sox2 at arginine 113. This methylation event enhances Sox2 self-association. Furthermore, the physiological retention of Sox2 on chromatin restricts the Sox2 methylation level. Our study reveals the direct regulation of Sox2 by CARM1 that sheds lights on how arginine methylation signals are integrated into the pluripotent transcription factor network.

## Introduction

As a member of the Sox (SRY-related HMG box) protein family, Sox2 is required for normal embryogenesis [1]. Embryonic stem cells (ESCs), derived from the inner cell mass of an early embryo, are capable of generating all the somatic cell types, and Sox2 is one of the core transcription factors that maintain the pluripotent state of ESCs [2]. Sox2 directly controls the expression of a large number of pluripotency-related genes, such as *oct3/4*, *nanog*, *fgf4* and *utf1* [3].

Sox family members are known to be tightly regulated by post-translational modifications [4]. Recent findings indicated that ubiquitination, sumoylation, acetylation and phosphorylation regulate Sox2 at multiple levels, including subcellular localization, DNA binding, and protein stability [5], [6], [7].

Among the various types of protein post-translational modifications, the methylation of arginine residues is catalyzed by a family of protein arginine methyltransferases (PRMTs) [8]. PRMT4/CARM1 catalyzes the asymmetric di-methylation of arginine residues in a variety of proteins, including histones and certain key transcription factors, and acts as a coactivator for transactivation [8]. CARM1 deficient mice are small in size, low in birth rate and die perinatally [9]. Moreover, overexpression of CARM1 at the two-cell-stage directs the blastomeres to contribute to the pluripotent inner cell mass, and the ESCs with depleted CARM1 gradually lose their pluripotency [10], [11]. However, the details describing how CARM1 modulates pluripotency maintenance and early embryonic development remain unclear.

In this work, we indicate that CARM1 facilitates Sox2-mediated transactivation and methylates Sox2 at Arg113. We also show that methylated Sox2 is more prone to self-associate than its unmethylated counterpart. In addition, the methylation level of Sox2 is restricted by the tight association of Sox2 with chromatin. Our data suggest a direct relationship between arginine methylation and Sox2 function.

## Results

### CARM1 is associated with Sox2

**Figure 1:**
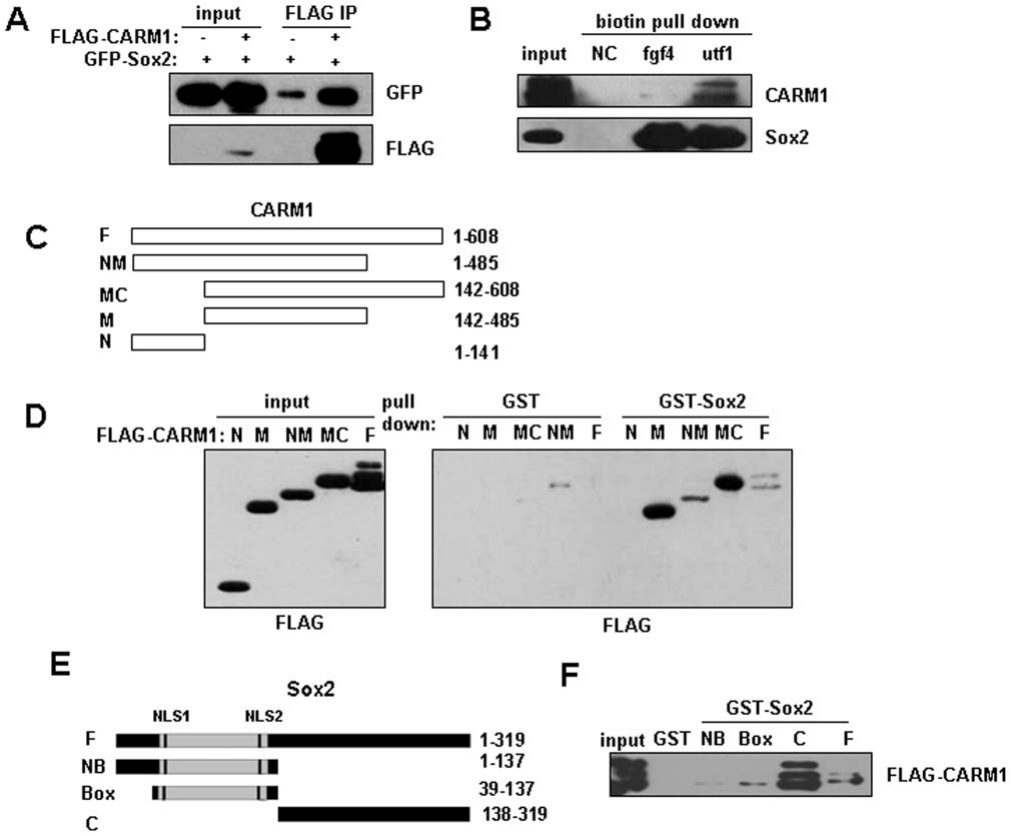
CARM1 is in association with Sox2. (A) Co-immunoprecipitation of Sox2 with CARM1. Whole cell extracts (WCEs) of MCF7 cells transfected with (+) or without (-) FLAG-CARM1 or GFP-Sox2, were subjected to immunoprecipitation with anti-FLAG antibody and blotted with antibodies against GFP or FLAG. (B) Co-precipitation of endogenous CARM1 with Sox2 by DNA pull-down. Biotin-labeled probes covering Sox2-binding sites in *fgf4* and *utf1* respectively were incubated with WCEs of P19 cells and streptavidin-conjugated agarose beads. The proteins precipitated with beads were denatured and resolved in SDS-PAGE and detected by immunoblotting with antibodies against Sox2 or CARM1. NC: negative control, no probe added. (C) Schematic drawing of the wild type and truncated CARM1 fragments. (D) GST pull-down assays to detect the interaction of Sox2 with CARM1 derivates. GST or GST-Sox2 was first incubated with WCEs of HEK293T cells ectopically expressing FLAG-CARM1 derivates. The GST pull-down products were immunoblotted with anti-FLAG antibody. Input: WCE control. (E) Schematic drawing of the wild type and truncated Sox2 fragments. (F) The interaction of Sox2 derivates with CARM1 in GST pull-down assays. GST or GST-Sox2 derivates were first incubated with whole cell extracts (WCEs) of HEK293T cells ectopically expressing FLAG-CARM1. The GST pull-down products were immunoblotted with anti-FLAG antibody.

Because CARM1 is required for the identity maintenance of ESCs, we supposed that CARM1 might directly regulate the core pluripotent transcription factor network. To verify this hypothesis, we first examined the interaction of CARM1 with Sox2, one of the most critical transcription factors during embryogenesis [1]. As shown in Fig. 1A, CARM1 can be co-immunoprecipitated by Sox2. Then we asked whether endogenous CARM1 is in association with Sox2. Both *fgf4* and *utf1* are typical Sox2 target genes [3], therefore, we used two biotin-labeled DNA probes, covering the Sox2-binding sites in *fgf4* and *utf1* respectively, to enrich endogenous Sox2 from P19 embryonal carcinoma cells [12], [13]. As indicated in Fig. 1B, endogenous Sox2 was sufficiently enriched by both DNA probes, and CARM1 can be co-precipitated. Next, we analyzed domain specificity of CARM1 to interact with Sox2. We generated GST-fused truncation proteins of CARM1. By performing GST pull-down assays, we found that the methyltransferase domain in the middle of CARM1 is efficient in mediating its interaction with Sox2 (Fig. 1C and D). Similarly, we found that both the HMG-box and C-terminal of Sox2 can mediate the interaction between Sox2 and CARM1 (Fig. 1E and F).

### CARM1 facilitates Sox2-mediated transactivation

**Figure 2:**
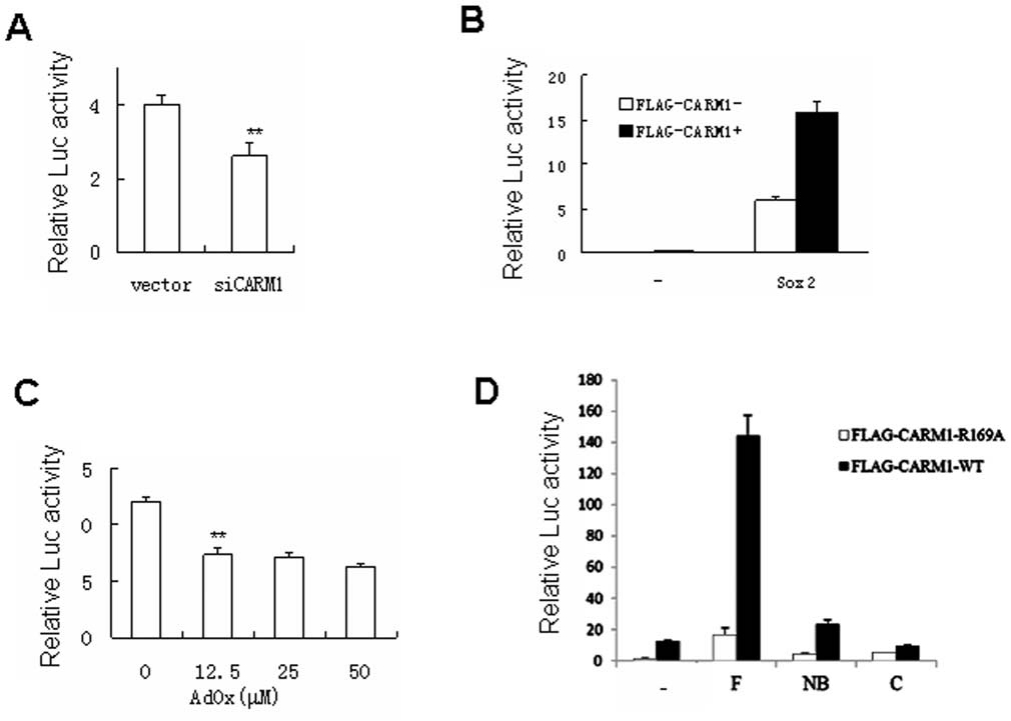
CARM1 facilitates Sox2 transactivation activity. (A) The effect of CARM1 knockdown on Sox2 mediated reporter activity assay. FLAG-Sox2 and the luciferase reporter plasmid (8×S/O-Luc) were co-transfected into MCF7 cells with either the plasmid of RNAi vector or siCARM1. (B) The effect of CARM1 overexpression on Sox2-mediated reporter activity. Wild-type Sox2 and its expression vector were individually transfected into MCF7 cells with or without FLAG-CARM1. (C) The effect of AdOx on Sox2-mediated reporter activity. MCF7 cells were first transfected with the 8×S/O-Luc reporter and FLAG-Sox2 for 24 hours. The cells were then treated with different concentrations of AdOx as indicated for another 24 hours before harvest for reporter activity assay. (D) The effect of CARM1-WT and CARM1-R169A overexpression on Sox2-mediated reporter activity. Wild type and truncated Sox2 (as shown in Fig. 1E) and its vector were individually transfected into MCF7 cells with CARM1-WT or CARM1-R169A. Each bar represents mean value ± S.D. from at least three independent experiments. ** *P*<0.01, Student's *t*-test.

To investigated the impact of CARM1 on Sox2-meditated transactivation, we constructed a Sox2 reporter plasmid 8×S/O-luc (Supporting information Figure S1), that can be efficiently activated by Sox2 [14]. CARM1 knockdown in MCF7 cells inhibited the reporter gene expression driven by Sox2 (Fig. 2A), while the overexpression of CARM1 dramatically enhanced the reporter activity (Fig. 2B). We also checked the reporter gene expression in MCF7 cells treated with a methyltransferase inhibitor adenosine dialdehyde (AdOx) and showed that inhibition of methylation reduced Sox2-mediated transactivation (Fig. 2C). In addition, we overexpressed CARM1 in pluripotent P19 cells, and found that several pluripotency-related genes were upregulated at the mRNA level (Supporting information Figure S2), while knockdown of CARM1 reduced the expression of these genes (Supporting information Figure S3). We next examined whether the facilitation of Sox2 transactivity by CARM1 relies on its catalytic activity. As reported that R169A substitution of CARM1 causes a complete loss of its methyltransferase activity [15], which is also confirmed in our 8×S/O-luc reporter assays that it was more active in the cells co-expressing Sox2 and wild-type CARM1 than those of Sox2 and CARM1 with R169A mutation (Fig. 2D).

### CARM1 methylates Sox2

**Figure 3:**
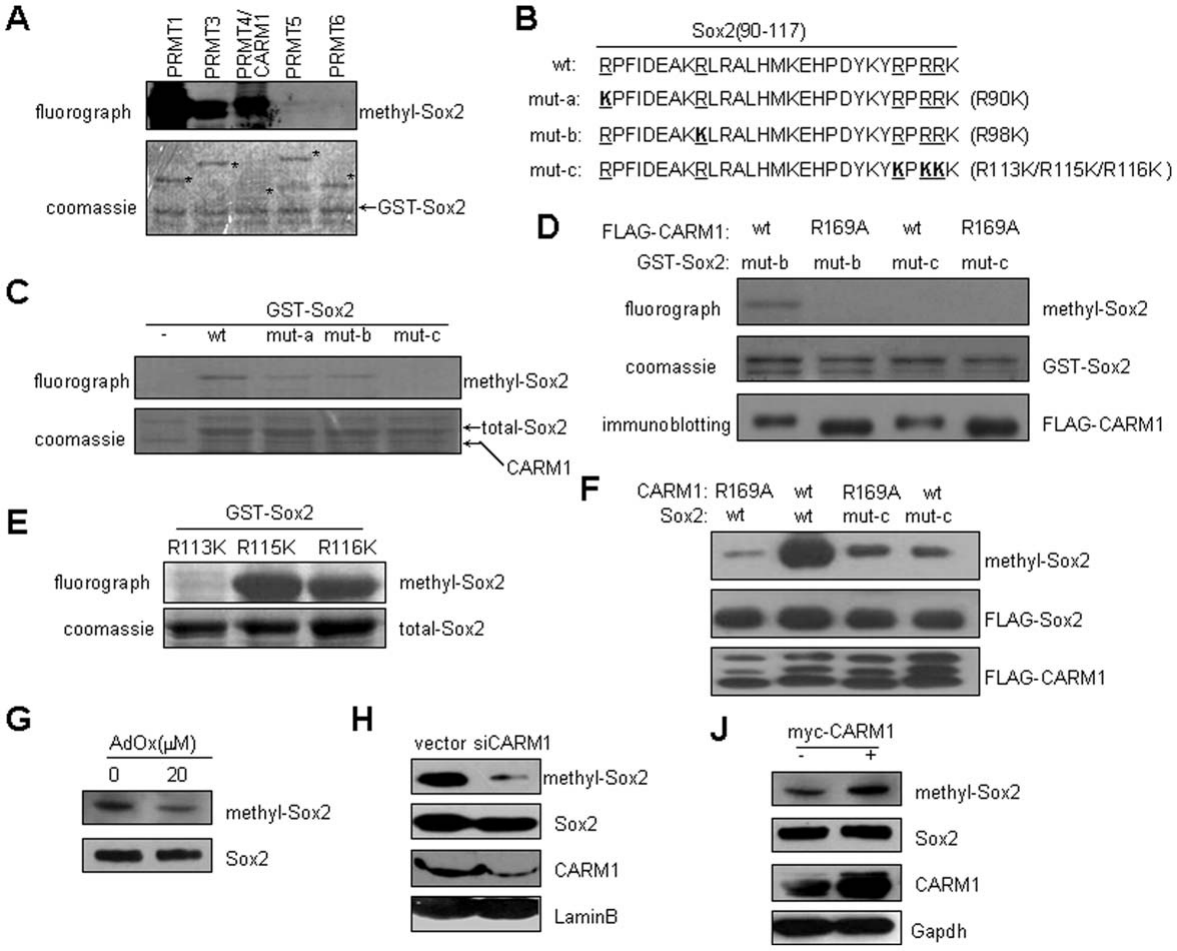
CARM1 methylates Sox2. (A) Methylation assays indicating methylation of Sox2 by PRMTs. Methylation signals were detected by fluorography. Total GST-Sox2 (arrowhead) and GST-PRMTs (asterisks) are shown by Coomassie brilliant blue staining. (B) The schematic representation of Sox2 methylation sites that are identified by mass spectrometry analysis. The sequence constitution of Sox2 mutants is shown, candidate methylation sites are underlined, and the substitutional lysine residues are in bold. (C and E) Methylation assays show Arg113 of Sox2 is the primary CARM1-methylated site. FLAG-tagged CARM1 was ectopically expressed in HEK293T cells and immunoprecipitated with anti-FLAG antibody. Equal amounts of FLAG-CARM1 immunoprecipitates were incubated with GST-Sox2 derivatives in the presence of [^3^H]-SAM. (D) Methylation assays indicating methylation of Sox2 by CARM1. FLAG-CARM1 and FLAG-CARM1-R169A were ectopically expressed in HEK293T cells and immunoprecipitated with anti-FLAG antibody. Equal amounts of FLAG-CARM1 and FLAG-CARM1-R169A immunoprecipitates were incubated with GST-Sox2 and GST-Sox2-mut-c in the presence of [^3^H]-SAM. Methylation signals were detected by fluorography. FLAG-CARM1 and FLAG-CARM1-R169A were detected with anti-FLAG antibody. Total GST-Sox2 or GST-Sox2-mut-c are shown by Coomassie brilliant blue staining. (F) Methylation of FLAG-Sox2 in MCF7 cells. Wild-type and mut-c of FLAG-Sox2 were overexpressed in MCF7 cells together with FLAG-CARM1 or FLAG-CARM1-R169A. Sox2 methylation at Arg113 was detected with anti-methyl-Sox2 antibody. (G) Methylation of endogenous Sox2 in P19 cells treated with AdOx. P19 cells were treated with AdOx for 24 hours. Sox2 methylation levels were detected with anti-methyl Sox2. (H) The effect of CARM1 knockdown with siCARM1 plasmid on Sox2 methylation in P19 cells. (J) The effect of CARM1 overexpression on Sox2 methylation in P19 cells. Myc-CARM1 (+) or Vector (-) were transfected into P19 cells and the Sox2 methylation levels were detected.

Because of the association of CARM1 with Sox2, we investigated whether Sox2 could be methylated by CARM1. As shown in Fig. 3A, Sox2 can be methylated by GST-tagged CARM1, and PRMT1, 3 and 5. Sox2 proteins methylated by PRMT1 and CARM1 were analyzed by mass spectrometry. Arginine residues Arg90, Arg98, Arg113 and Arg115 were identified as target sites for PRMT1, and Arg113, Arg115, and Arg116 were identified for CARM1 (Fig. 3B and Supporting information Figure S4). To confirm the mass spectrometry data, these five arginine residues were mutated to lysine (R to K) individually or in combination (Fig. 3B). In addition, FLAG-tagged PRMTs, immunoprecipitated from the whole cell extracts of HEK293T cells, were used to mimic their respective physiological substrate specificity. We found that FLAG-PRMT4/CARM1 methylated GST-Sox2 and the methylation level was only slightly reduced by a single mutation of Sox2 at Arg90 (mut-a) or at Arg98 (mut-b), however, Sox2 methylation was abolished by combined substitution of Arg113, Arg115, and Arg116 (mut-c) (Fig. 3C). Moreover, we found that immunoprecipitated CARM1 mutants were unable to methylate Sox2 at all, we thereby conclude that the methylation of Sox2 by CARM1 is dependent on the catalytic activity of CARM1 (Fig. 3D). By the respective substitutions to lysine, we demonstrated that Arg113, but not Arg115 or Arg116, is crucial for CARM1-mediated Sox2 methylation (Fig. 3E). These data suggest that CARM1 methylates Sox2 preferentially at Arg113.

To detect whether Sox2 is methylated physiologically, we raised a polyclonal antibody against methylated Sox2 in rabbit. This antibody specifically recognized the Sox2 peptide asymmetrically di-methylated at Arg113, but not the unmethylated Sox2 peptide (Supporting information Figure S5). By co-expression of Sox2 with CARM1 in MCF7 cells, we found that the recognition of wild-type Sox2 by the anti-methyl-Sox2 antibody was promoted by enzymatically active CARM1. In contrast, CARM1 did not enhance the methylation level of Sox2 with combined mutations of R113/R115/R116 (Fig. 3F).

Next, we treated P19 cells with AdOx to investigate its effect on the methylation of endogenous Sox2. AdOx treatment clearly reduced the recognition of endogenous Sox2 by the anti-methyl-Sox2 antibody, we thereby deduced that endogenous Sox2 is methylated at Arg113 (Fig. 3G). To explore whether CARM1 accounts for the methylation of endogenous Sox2, CARM1 was knocked down or overexpressed in P19 cells, and Sox2 methylation level was detected. As shown in Fig. 3H, while CARM1 depletion led to a reduction in Sox2 methylation, its overexpression was accompanied by an increased methylation level on Sox2 (Fig. 3J). These data indicate that CARM1-mediated the methylation of Arg113 in Sox2.

### The impact of CARM1-mediated methylation on Sox2 self-association

**Figure 4:**
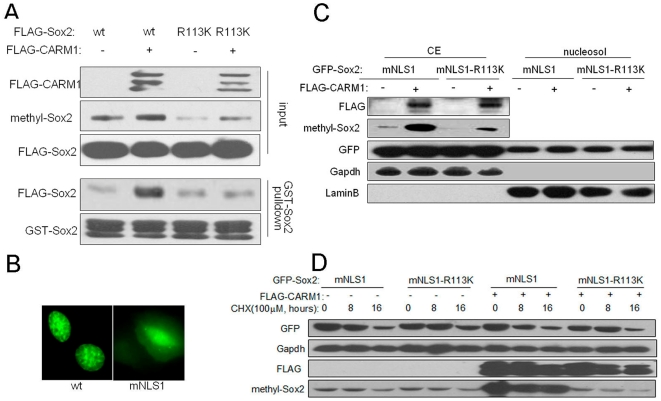
The effects of Arg113 methylation on Sox2 self-association, subcellular localization and protein stability. (A) The effect of CARM1-mediated Sox2 methylation on Sox2 self-association. FLAG-Sox2 wild-type and mutant-c were expressed together with (+) or without (-) FLAG-CARM1 in MCF7 cells. WCEs were immunoblotted to examine the Sox2 methylation level. The remaining lysates were subjected to pull-down assays with bacterially expressed GST-Sox2 protein. (B) Localization of wild-type and R113K mutant GFP-tagged Sox2 in MCF7 cells. (C) The impact of CARM1-mediated methylation on the GFP-Sox2-mNLS1 distribution in cell fractions. GFP-tagged Sox2-mNLS1 with or without R113K substitution was expressed in MCF7 cells, and cell fractions were prepared to detect Sox2 distribution. CE: cytoplasmic extracts; nucleosol: soluble nuclear materials. (D) The effect of CARM1 on GFP-Sox2-mNLS1 protein stability. GFP-Sox2-mNLS1 and GFP-Sox2-mNLS1-R113K were expressed in MCF7 cells to examine the effect of CARM1 on Sox2 degradation. Forty-eight hours later, cells were treated with cycloheximide (CHX) as indicated, and Sox2 protein levels were analyzed by immunoblotting assays.

It has been reported that several Sox proteins form homo- or heterodimer complexes that lead to different functional outputs and that Sox2 can self-associate through its HMG-box [16]. Here, we examined the effect of methylation on Sox2 self-association. As indicated in Fig. 4A, co-expression of wild-type Sox2 with CARM1 not only increased Sox2 methylation, but also promoted the interaction between FLAG-Sox2 and GST-Sox2. However, the affinity of FLAG-Sox2-mut-c to GST-Sox2 was less sensitive to the co-existence of CARM1.

Sox2 possesses two nuclear localization signals [17] and residue Arg113 is within NLS2 (Supporting information Figure S6). Therefore, we postulated that CARM1-mediated Sox2 methylation is related to NLS2 function. To augment this assumed modulation of NLS2 by methylation, Sox2 NLS1 was perturbed (mNLS1). Sox2-mNLS1 had an obvious distribution in the cytoplasm, whereas wild-type Sox2 localized to the nucleus predominantly (Fig. 4B). By analyzing the Sox2 distribution in subcellular fractions, we found that neither the R113K mutation nor the co-expression of CARM1 had obvious effect on the subcellular localization of GFP-Sox2-mNLS1 (Fig. 4C).

We also explored whether CARM1 affects Sox2 protein stability. GFP-Sox2-mNLS1 was expressed alone or in combination with CARM1 in MCF7 cells, and the cells were treated with cycloheximide (CHX) to inhibit *de novo* protein synthesis. As indicated in Fig. 4D, overexpression of CARM1 enhanced Sox2 methylation with no obvious change on its degradation.

These results suggest that Arg113 methylation promotes Sox2 self-association but does not affect Sox2 subcellular localization or degradation.

### The role of NLS1 in Sox2 methylation

**Figure 5:**
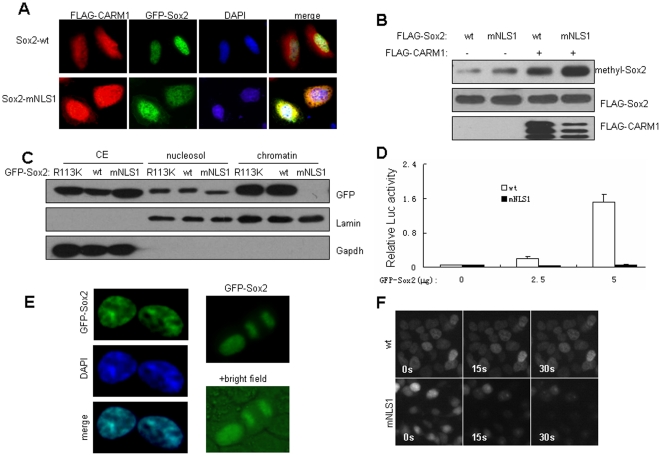
The role of NLS1 in CARM1-mediated Sox2 methylation. (A) Wild-type and mNLS1 of GFP-Sox2 were overexpressed in MCF7 cells together with FLAG-CARM1. Red, CARM1 immunostained with anti-FLAG antibody; green, GFP-Sox2; blue, DNA. Upper panel: GFP-Sox2-wt; lower panel: GFP-Sox2-mNLS1. (B) The impact of the NLS1 mutation on Sox2 methylation. (C) The distribution of Sox2 derivatives in MCF7 cell fractions. CE: cytoplasmic extracts; nucleosol: soluble nuclear materials; chromatin: extracts from unsoluble chromatin pellets with 500 mM NaCl. (D) Transactivation activity of GFP-Sox2-mNLS1 in MCF7 cells. The wild-type GFP-Sox2 was used as a control. ** *P*<0.01, Student's *t*-test. (E) Tight association of Sox2 with chromatin in MCF7 cells. Left: The subcellular localization of wild-type GFP-Sox2; right: GFP-Sox2 bound to chromosomes in mitosis (picture at the bottom indicates the overlap of GFP-Sox2 and the background under bright field microscopy). (F) Chromatin retention of GFP-Sox2. MCF7 cells expressing GFP-Sox2-wt or GFP- Sox2-mNLS1 were permeabilized with 0.3% NP-40. Sequential images were captured before and after permeabilization.

Because CARM1 localizes primarily to the cytoplasm and Sox2-mNLS1 displays an increased co-localization with CARM1 in MCF7 cells (Fig. 5A), which inspired us to examine whether Sox2 methylation level is affected by NLS1 mutation. Indeed, perturbation of NLS1 significantly enhanced Sox2 methylation (Fig. 5B). Unexpectedly, we found that NLS1 dysfunction abolished the chromatin association of Sox2 (Fig. 5C). Consistently, Sox2-mNLS1 completely lost the ability to activate the 8×S/O-luc reporter (Fig. 5D). Wild-type Sox2 stringently co-localizes with DNA (Fig. 5E). After cells were permeabilized as described [18], wild-type Sox2 was still tightly attached to chromatin with no obvious diffusion; however, most of the Sox2-mNLS1 proteins diffused out of the cells in less than thirty seconds (Fig. 5F). Therefore, the physiological retention of Sox2 on chromatin restricts its free movement, and might consequently limit its nuclear-cytoplasm exchange and contact with CARM1. These results suggested that the chromatin retention of Sox2 restricts CARM1-mediated Sox2 methylation.

## Discussion

In this work we demonstrate that CARM1 is in association with Sox2 and facilitates Sox2-mediated transactivation. We identify Sox2 as a novel substrate for CARM1. Methylated Sox2 has increased self-association, but displays no obvious change in protein stability or subcellular localization. However, the Sox2 methylation level is restricted by the tight association of Sox2 with chromatin.

CARM1 has been shown to act as a coactivator of a number of transcription factors through direct modification of histone arginines, and subsequently, it increases local chromatin accessibility for transcriptional machinery [19]. This impelled us to assume that Sox2 might also recruit CARM1 to its target genes to modulate the chromatin architecture. Consistent with this hypothesis, histones methylated at H3R17 and/or H3R26 are especially enriched at the promoters of several key Sox2 target genes in ESCs [11]. Indeed, our data indicate that CARM1 interacts with Sox2 and enhances Sox2-mediated transactivation.

The structure of the HMG box and the residues flanking methylation site of Sox2 are conserved among Sox family members (Supporting information Figure S7) [20]. Recently, CARM1 was reported to methylate Sox9, a Sox family member implicated in chondrogenesis and sex determination [21]. Therefore, it is reasonable to hypothesize that the CARM1-mediated arginine methylation is a prevalent modulation mechanism throughout the Sox family.

Sox2, Oct3/4 and Nanog co-occupy a series of target genes that are discriminately expressed in ESCs [2]. Moreover, Sox2 plays indispensable roles at multiple stages of embryogenesis [22]. Consistently, Sox2 modulates different target gene groups by synergistic action with distinct partners [23]. Given the increased self-association of methylated Sox2, it would be interesting to identify protein profiles that associate with Sox2, and particularly with methylated Sox2, at different developmental stages.

Another key pluripotent transcription factor, Oct3/4, is also subjected to multiple post-translational modifications [24]. Due to the essential role of PRMTs in embryonic development [25], it is reasonable to suggest that numerous transcription factors and regulatory proteins involved in pluripotency maintenance are potential targets for arginine methylation. We expect that the further proteomic identification of PRMT-associated proteins and arginine methylation substrates in pluripotent cells would shed light on the mechanism of PRMTs-mediated modulation of early embryogenesis.

## Materials and Methods

### Cell culture and treatment

Human breast cell line MCF7 and mouse embryonal carcinoma cell line P19 were purchased from Cell Resource Center, IBMS, CAMS/PUMC. MCF7 cells were maintained in DMEM containing 10% fetal calf serum. P19 embryonal carcinoma cells were maintained in alpha MEM supplemented with 10% fetal calf serum. Differentiation of P19 cells was induced with 0.5 µM of retinoic acid as described [13].

### Plasmid, constructs, transfections and Reporter Gene Assays

To obtain the 8×(S/O)-luciferase reporter construct, four copies of annealed oligonucleotides containing two *fgf*-4 enhancer elements [14] were tandemly inserted into the pGL3-promoter plasmid digested with XhoI and NotI, as indicated in Supporting information Figure S1. Site-directed mutagenesis of the Sox2 expression plasmids was conducted according to Stratagene's protocol (Stratagene QuikChange Site-directed Mutagenesis kit). The FLAG-CARM1 expression plasmid was a gift of Dr. Xu Wei. The CARM1 RNAi construct siCARM1 was generated by subcloning double-stranded oligomers into the *Bbs*I digested pBabe-Dual vector [26] (forward 5′-AAAGTCCAGTAACCTCCTGGAT-3′; reverse 5′-AAAAATCCAGGAGGTTACTGGA-3′). The Sox2 coding sequence was subcloned into EGFP-C1 to construct GFP-Sox2. Myc-CARM1 was constructed by the insertion of the CARM1 CDS into pCMV-tag-3B.

Cells were transfected using the Vigofect reagent (Vigorous, China) in accordance with the manufacturer's instructions.

For the firefly luciferase activity analysis, the CMV-Renilla plasmid was used as an internal control to normalize for transfection efficiency. The statistical significance (P value) was calculated by unpaired Student's *t*-test.

For CARM1 knock down in P19 cells, nineteen base-pair of oligo-RNA segments (sense, 5′-GGAUAGAAAUCCCAUUCAAdTdT-3′, antisense, 5′-UUGAAUGGGAUUUCUAUCCdTdT-3′) were transfected with Lipofectamine 2000 (Invitrogen) into P19 cells. The cells were harvested after 36 hours for RNA extraction.

### Quantitative Real-Time RT-PCR analysis

Total RNA was purified with Trizol reagent (Invitrogen) according to the manufacturer's instructions. One microgram of total RNA was used for cDNA synthesis with a Reverse transcription kit (Invitrogen). Quantitative real-time PCR was performed using the ExTaq Mix kit (Takara), and the primer sequences used are listed as follow: (F; forward; R; reverse.)

Sox2, F: TCCATGACCAGCTCGCAGA; R: GAGGAAGAGGTAACCACGGG

Oct3/4, F: TCACTCACATCGCCAATCA; R: GTAGCCTCATACTCTTCTCGTT

Nanog, F: GGCAGCCCTGATTCTTCTAC; R: CGCTTGCACTTCATCCTTT

Lin28, F: CGGCCAAAAGGGAAGAACAT; R: CATTCCTTGGCATGATGGTCT

Fgf4, F: GGCTTCGGCGGCTCTACT; R: AGGATTCGTAGGCGTTGTAGTTG

Utf1, F: TCCTCTTACGAGCACCGACAC; R: GCAACGCGGTATTCAACGA

CARM1, F: TGTGGCTGGAATGCCTACTG; R: TTGGACAATGCCCGTGCT

GAPDH, F: GAAGGTGAAGGTCGGAGTC; R: GAAGATGGTGATGGGATTT

### Preparation of GST-fusion proteins, methylation assays and mass spectrometry analysis

Protein coding sequences of PRMTs and Sox2 were inserted into the pGEX-6p-1 plasmid individually, and GST-fusion proteins were expressed in *E. coli* BL21 and purified as per Pharmacia's instructions. Methylation assays were performed as described [27]. Briefly, each GST-PRMT was mixed with its respective substrate in 1x methyltransferase buffer (50 mM Tris [pH 8.0], 1 mM PMSF, 0.5 mM DTT) along with 1 µCi of [^3^H]S-adenosylmethionine (SAM) or unlabeled 100 µM SAM and brought up to a total reaction volume of 50 µl. Reactions were incubated at 30°C for one hour. Samples were resolved by SDS-PAGE and stained with Coomassie brilliant blue. After destaining, gels with radioactively labeled samples were visualized by fuorography with EN^3^HANCE (PerkinElmer) according to the manufacturer's instructions. Gel bands corresponding to methylated substrates with unlabeled SAM were excised, cut into small cubes, digested in-gel with trypsin and subjected to liquid chromatography-tandem mass spectrometry (LC-MS/MS) analysis (IBP, CAS, Beijing). MS/MS spectra were analyzed using Bioworks 3.1 software.

### Preparation of cell fractions

Fractions of MCF7 cells were prepared as previously described [28] and partially modified according to Steven Henikoff lab's protocol [29]. Briefly, to collect the cytoplasmic extraction, cells were resuspended in 1× buffer A (10 mM HEPES, pH 7.9, 1.5 mM MgCl_2_, 10 mM KCl, 0.5 mM DTT, 1 mM PMSF, 10 µg/ml aprotinin, 10 µg/ml leupeptin) and left on ice for 5 minutes. NP-40 was added to 0.3%, and samples were immediately pipetted up and down to disrupt cell membranes. Samples were centrifuged at 500 g for 5 min at 4°C. Supernatants were then centrifuged at maximum speed (16,000 g) for 15 min at 4°C, and supernatants were stored as Cytoplasmic Extract ‘CE’. Nuclei pellets obtained as above were then lysed in 500 µl RIPA buffer (50 mM HEPES, pH 7.5, 150 nM NaCl, 2 mM EDTA, 2 mM EGTA, 1% TritonX-100, 50 mM NaF, 5 mM sodium pyrophosphate, 50 mM sodium β-glycerophosphate, 1 mM sodium ortho-vanadate, 1 mM DTT, 1 mM PMSF, 10 µg/ml leupeptin, 10 µg/ml aprotinin) for 30 minutes with gentle rotation. Samples were centrifuged at maximum speed (16,000 g) for 15 min at 4°C, and the supernatants were stored as ‘nucleosol’, mainly containing the physiologically soluble nuclear material. To obtain the ‘chromatin’ fraction, the pellets from above were washed twice with RIPA buffer, then resuspended in 500 µl RIPA buffer (containing protease inhibitors) with NaCl up to 500 mM, and extracted for an hour. The supernatants were then collected.

### GST Pull-down, co-immunoprecipitation, immunoblotting assays and immunostaining

GST Pull-down, co-immunoprecipitation, immunoblotting and immunostaining assays were carried out as previously described [30] with a partial modification. When detecting the interaction between Sox2 and CARM1, we used 0.2% NP-40 to lyse cells and wash agarose beads.

### Cell permeabilization assays

Cell permeabilization assays were conducted as previously described [18].

### DNA pull-down assays

DNA pull-down assays were performed as previously described [31]. In brief, 50 nmol 5′-end biotin-labeled oligo-DNA probes covering the Sox2-binding sites in *fgf4* or *utf1* were incubated with 1 mg whole cell extracts of P19 cells and streptavidin-conjugated agarose beads (Millipore) overnight at 4°C. Then the beads were collected and washed five times with lysis buffer and prepared for SDS-PAGE and detected by immunoblotting. The sequences of oligo-DNA are indicated as below:

fgf4, sense strand:

biotin-CTGAAAGAAAACTCTTTGTTTGGATGCTAATGGGATACTAAGCTGA;

antisense strand:

biotin-TCAGCTTAGTATCCCATTAGCATCCAAACAAAGAGTTTTCTTTCAG.

utf1, sense strand:

biotin-CTGAAAGATGAGAGCCCTCATTGTTATGCTAGTGAAGTGCCAAGCTGA;

antisense strand:

biotin-TCAGCTTGGCACTTCACTAGCATAACAATGAGGGCTCTCATCTTTCAG.

### Antibodies

Antibodies are listed as follows: anti-GFP (MBL, M048-3), anti-CARM1 (CST, 4438s), anti-FLAG (Sigma, F3165), anti-Gapdh (Chemicon, MAB374), anti-Lamin-B (Santa Cruz, sc-6216), and anti-Sox2 (Chemicon, AB5603). A polyclonal rabbit antibody against di-methylated Arg113 of Sox2 (anti-methyl-Sox2) was raised using the methylated peptide KEHPDYXYR_di-methyl_PRRKTKC (asymmetric di-methylarginines are underlined) and purified using a peptide-affinity column.

## Supporting Information

Figure S1 Schematic representation of 8×S/O-luc reporter plasmid. 8×S/O-luc carries eight copies of tandem fgf4 enhancer elements consisting of neighboring binding sites for Sox2 and Oct3/4.
(JPG)

Figure S2 The effect of CARM1 overexpression on the expression of pluripotency-related genes in P19 cells. The relative mRNA levels of pluripotency markers were normalized to Gapdh.
(JPG)

Figure S3 The effect of CARM1 knockdown with synthesized siRNA on the expression of pluripotency-related genes in P19 cells. The relative mRNA levels of pluripotency markers were normalized to Gapdh. Control: scrambled siRNA.
(JPG)

Figure S4 MS/MS spectrum of the Sox2 peptide EHPDYKYRdi-methylPR. GST-Sox2 methylated by GST-CARM1 was digested with trypsin and subjected to LC-MS/MS analysis. The MS/MS spectrum of a peptide corresponding to residues 106-115 of Sox2 showed that Arg113 was di-methylated. An asterisk indicates the methylated arginine.
(JPG)

Figure S5 Specifically recognition of Sox2 peptide methylated at Arg113, by anti-methyl-Sox2 antibody. Un-methylated peptide KEHPDYXYRPRRKTKC (Arg113 is underlined) designated as ‘R113me0’, and the peptide asymmetrically di-methylated at Arg113 named as ‘R113me2’ were used in dot blot analysis.
(JPG)

Figure S6 Schematic representation of Sox2 nuclear localization signals. The key residues of NLSs are underlined, and an asterisk indicates the identified methylation site Arg113. The residues that are mutated in Sox2-mNLS1 are also shown.
(JPG)

Figure S7 CARM1-mediated methylation may be a regulation mechanism shared by certain other Sox family members. Alignment of Sox members highly conserved in sequences flanking Arg113 was shown. NLS2 was underlined, Arg113 and its counterparts in other Sox proteins were pointed out with asterisks, conserved residues were indicated in shadow.
(JPG)
