# Supplementary figures and images for: CARM1 Mediates Modulation of Sox2
Source: PLoS One. 2011 Oct 28;6(10):e27026. doi: 10.1371/journal.pone.0027026 (PMC3203945; doi:10.1371/journal.pone.0027026)

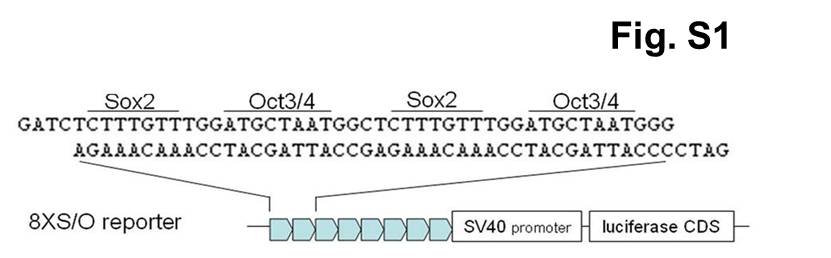

Supplement: Figure S1 — Schematic representation of 8×S/O-luc reporter plasmid. 8×S/O-luc carries eight copies of tandem fgf4 enhancer elements consisting of neighboring binding sites for Sox2 and Oct3/4. (JPG) [file pone.0027026.s001.jpg]

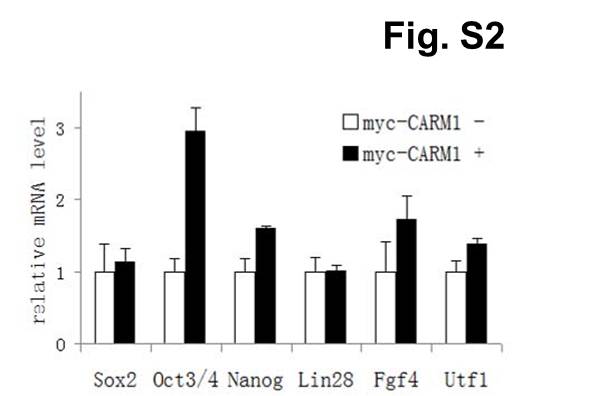

Supplement: Figure S2 — The effect of CARM1 overexpression on the expression of pluripotency-related genes in P19 cells. The relative mRNA levels of pluripotency markers were normalized to Gapdh. (JPG) [file pone.0027026.s002.jpg]

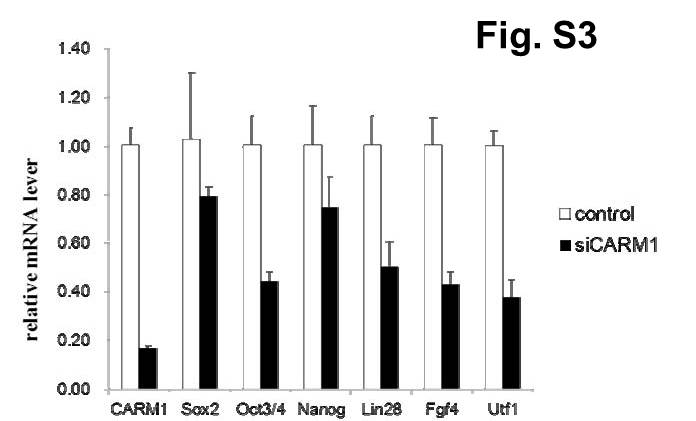

Supplement: Figure S3 — The effect of CARM1 knockdown with synthesized siRNA on the expression of pluripotency-related genes in P19 cells. The relative mRNA levels of pluripotency markers were normalized to Gapdh. Control: scrambled siRNA. (JPG) [file pone.0027026.s003.jpg]

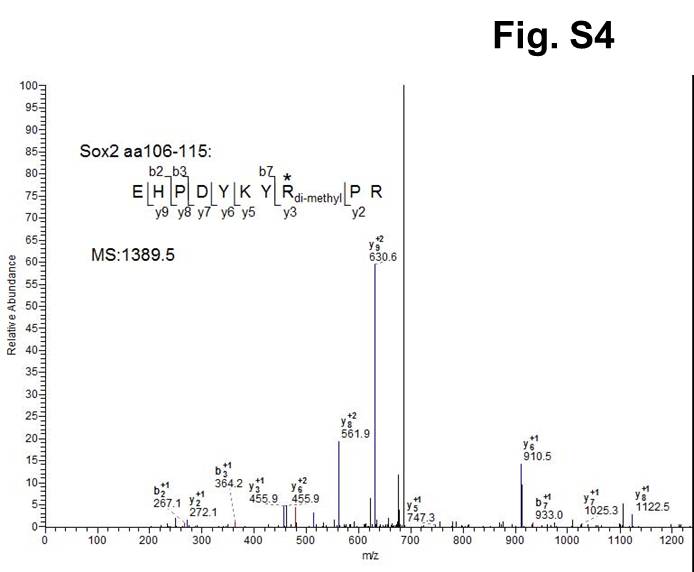

Supplement: Figure S4 — MS/MS spectrum of the Sox2 peptide EHPDYKYRdi-methylPR. GST-Sox2 methylated by GST-CARM1 was digested with trypsin and subjected to LC-MS/MS analysis. The MS/MS spectrum of a peptide corresponding to residues 106-115 of Sox2 showed that Arg113 was di-methylated. An asterisk indicates the methylated arginine. (JPG) [file pone.0027026.s004.jpg]

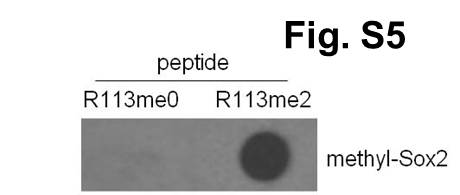

Supplement: Figure S5 — Specifically recognition of Sox2 peptide methylated at Arg113, by anti-methyl-Sox2 antibody. Un-methylated peptide KEHPDYXYRPRRKTKC (Arg113 is underlined) designated as ‘R113me0’, and the peptide asymmetrically di-methylated at Arg113 named as ‘R113me2’ were used in dot blot analysis. (JPG) [file pone.0027026.s005.jpg]

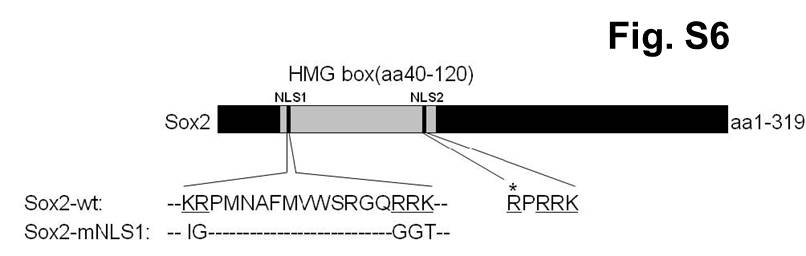

Supplement: Figure S6 — Schematic representation of Sox2 nuclear localization signals. The key residues of NLSs are underlined, and an asterisk indicates the identified methylation site Arg113. The residues that are mutated in Sox2-mNLS1 are also shown. (JPG) [file pone.0027026.s006.jpg]

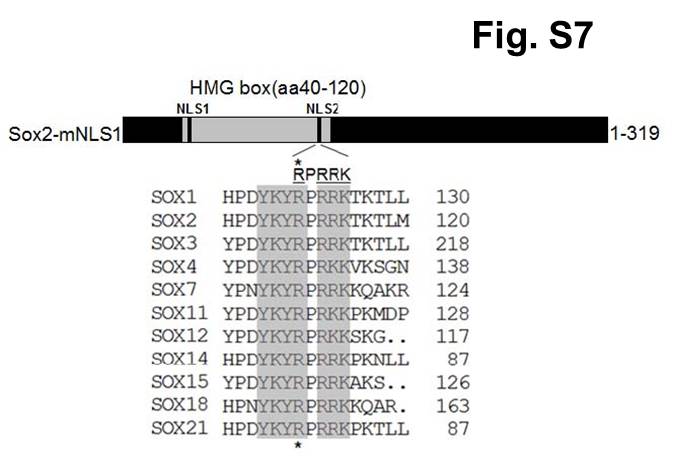

Supplement: Figure S7 — CARM1-mediated methylation may be a regulation mechanism shared by certain other Sox family members. Alignment of Sox members highly conserved in sequences flanking Arg113 was shown. NLS2 was underlined, Arg113 and its counterparts in other Sox proteins were pointed out with asterisks, conserved residues were indicated in shadow. (JPG) [file pone.0027026.s007.jpg]
